# Cancer multidisciplinary team meetings: impact of logistical challenges on communication and decision-making

**DOI:** 10.1093/bjsopen/zrac093

**Published:** 2022-08-27

**Authors:** Tayana Soukup, Benjamin W Lamb, Abigail Morbi, Nisha J Shah, Anish Bali, Viren Asher, Tasha Gandamihardja, Pasquale Giordano, Ara Darzi, Nick Sevdalis, James S A Green

**Affiliations:** Centre for Implementation Science, Health Service & Population Research Department, King's College London, London, UK; Department of Urology, Cambridge University Hospital NHS Trust, London, UK; Department of Surgery and Cancer, Imperial College London, London, UK; HeLEX Centre, University of Oxford, Oxford, UK; Royal Derby Hospital, Derby, UK; Royal Derby Hospital, Derby, UK; Chelmsford Breast Unit, Broomfield Hospital, Chelmsford, UK; Whipps Cross University Hospital, Barts Health NHS Trust, London, UK; Department of Surgery and Cancer, Imperial College London, London, UK; Centre for Implementation Science, Health Service & Population Research Department, King's College London, London, UK; Whipps Cross University Hospital, Barts Health NHS Trust, London, UK

## Abstract

**Background:**

Multidisciplinary teams (MDTs) are widely used in cancer care. Recent research points to logistical challenges impeding MDT decision-making and dissatisfaction among members. This study sought to identify different types of logistical issues and how they impacted team processes.

**Methods:**

This was a secondary analysis of a cross-sectional observational study. Three cancer MDTs (breast, colorectal, and gynaecological) were recruited from UK hospitals. Validated observational instruments were used to measure decision-making (Metrics of Observational Decision-making, MDT-MODe), communication (Bales' Interaction Process Analysis, Bales' IPA), and case complexity (Measure of Case Discussion Complexity, MeDiC), including logistical challenges (Measure of Case Discussion Complexity, MeDiC), across 822 case discussions from 30 videoed meetings. Descriptive analysis and paired samples *t* tests were used to identify and compare frequency of different types of logistical challenges, along with partial correlations, controlling for clinical complexity of cases, to understand how such issues related to the MDT decision-making and communication.

**Results:**

A significantly higher frequency of administrative and process issues (affecting 30 per cent of cases) was seen compared with the frequency of equipment issues (affecting 5 per cent of cases; *P* < 0.001) and the frequency of the attendance issues (affecting 16 per cent of cases; *P* < 0.001). The frequency of the attendance issues was significantly higher than the frequency of equipment issues (*P* < 0.001). Partial correlation analysis revealed that administrative and process issues, including attendance, were negatively correlated with quality of information (*r* = −0.15, *P* < 0.001; *r* = −0.11, *P* < 0.001), and equipment issues with the quality of contribution to meeting discussion (*r* = −0.14, *P* < 0.001). More questioning and answering by MDT members was evident with the administrative and process issues (*r* = 0.21, *P* < 0.001; *r* = 0.19, *P* < 0.001). Some differences were observed in teams' socioemotional reactions to the administrative and process issues with the gynaecological MDT showing positive correlation with positive socioemotional reactions (*r* = 0.20, *P* < 0.001), and the breast cancer MDT with negative socioemotional reactions (*r* = 0.17, *P* < 0.001).

**Conclusion:**

Administrative and process issues were the most frequent logistical challenges for the studied teams. Where diagnostic results were unavailable, and inadequate patient details provided, the quality of decision-making was reduced.

## Teaser

Multidisciplinary teams (MDTs) are a standard in cancer care. We found that logistical challenges in MDT meetings impede team decision-making and communication processes. This has implications for team efficiency, as well as quality.

## Introduction

A multidisciplinary approach is widely used in the management of patients with cancer^1–5^. A team typically includes histopathologists, radiologists, surgeons, cancer nurse specialists (CNSs), and oncologists, in typically weekly or fortnightly meetings (sometimes described as ‘tumour boards’). Medical history and test results are reviewed, and treatment options are formulated. This process involves new patients, those undergoing staging procedures to clarify management, and those undergoing treatment^1–5^.

Evidence around the effectiveness of the multidisciplinary approach to cancer care has been widely examined actively^6–15^. The pattern of decision-making suggests unequal participation in discussion and suboptimal sharing of information, which can affect the ability of the team to reach a treatment recommendation along with its implementataion^6–15^. Multidisciplinary teams (MDTs) are also affected by the changing economic/political landscape surrounding healthcare^16,17^, cancer incidence^16,18^, staff shortages^19^, workload^20^, and a variety of local factors such as availability of time, number of cases for discussion, case complexity, team size, sex composition, and disciplinary diversity^21–25^.

Little is known, however, about the impact of logistics that support the MDT meetings on decision-making. Two recent studies identified administrative and process issues, attendance, and issues with the meeting equipment to be relevant, with a significant reduction in the quality of decision-making and an increase in negative reactions, such as disagreements and antagonism. More than 40 per cent of cases discussed at the MDT meetings encountered a logistical problem^24,25^.

Unpacking the logistical challenges that arise in MDT meetings is important to improve an understanding of how frequently they occur and how they relate to decision-making and communication^24–26^. The aim of the study was to explore the frequency of logistical challenges among cases discussed by the MDT and understand the relationship between these issues and the quality of decision-making and communication in MDT meetings.

## Methods

The STROBE checklist was followed (*Table S1*)^27^.

### Study design and setting

This was a secondary analysis of an existing data set, used in a cross-sectional observational study^28,29^. The study took place across three university hospitals in the Greater London and Derbyshire areas in the UK between September 2015 and July 2016. Three cancer MDTs took part, including breast, colorectal, and gynaecological cancers; each team was from a different hospital. Each participating MDT had 10 of their weekly meetings video recorded. The study was granted ethical and regulatory approvals by the North West London Research Ethics Committee (JRCO ref. 157441), and locally by R&D departments of the participating NHS Trusts. Informed consent was sought from all participants. The study was adopted by the National Institute for Health Research Clinical Research Network Portfolio.

### Participants and sample size

A detailed breakdown of the team composition has been published previously^23^. All cases on the agenda for discussion were video recorded. Sample size needed to detect significance was estimated to be 396 case discussions (Pearson correlations calculated using G*Power 3 for *a priori* power analysis with *d* = 0.50; *α* = 0.05; and 1 − *β* = 0.95). Availability sampling was used to identify the teams with a criterion for the study being a cancer MDT from the UK National Health Service (NHS) dealing with three common types of cancer.

### Statistical analyses

Quantitative observational assessments were conducted for each case discussion using three validated observational instruments: Metrics for Observation of Decision-making (MDT-MODe) for the assessment of MDT decision-making^9–12^, Bales' Interaction Process Analysis (Bales' IPA) for MDT communication and interaction^30,31^, and Measure of Case Discussion Complexity (MeDiC) for clinical and logistical complexities of the cases^32,33^ (*Tables S2* and *S3*). All assessments were conducted by assessment of the video recordings.

Training in the use of the three observational tools was undertaken by all evaluators before the formal scoring during the study. Proficiency in scoring was set as an achievement of inter-assessor reliability of 0.70 or higher between the trainee and expert assessor^34^ across all three observational instruments using interclass correlation coefficients (ICCs). Second assessors rated 15–20 per cent of case discussions for each tool respectively, and their scores calibrated against the main assessor. For Bales' IPA, scores were calibrated with a social scientist; for MDT-MODe with an academic consultant surgeon; and for MeDiC with an academic physician. Each evaluator was blind to the other evaluators' observations.

Observer bias was addressed and reliability of evaluations on the three instruments was ensured by having a subset of cases scored by the evaluators in pairs who were all trained in the use of the instruments. During data collection, each evaluator was blind to the other evaluators' observations. To reduce the Hawthorne effect, where teams might change their usual behaviour due to being observed, a long-term approach was used by filming each team for a prolonged time (3 months/12 consecutive weeks), where the first two meetings for each team were excluded from the analysis. A small recording camera with sound settings and recording light switched off, and remote control to start and stop recording was used with the camera positioned to blend in with background equipment and cables, out of immediate view of the team.

Logistical challenges were identified from the video recordings using the MeDiC tool and analysed and interpreted using a thematic approach where a systematic classification process of coding and identifying patterns in the data were applied, as published previously^29,32^.

To gauge frequency of logistical challenges across the studied cases, descriptive and frequency analyses were conducted on the identified types of logistical challenges (administrative and process issues, attendance, and equipment issues). Paired samples *t* tests were then used to explore differences in the frequency of the individual logistical challenges.

To examine the relationship between logistics and team processes, partial correlation analyses between MDT-MODe (decision-making)^9–12^, Bales' IPA (communication)^30,31^, and the identified logistical challenges (administrative and process issues, attendance, and equipment issues) were used, controlling for clinical case complexity using MeDiC^32,33^. All pairwise comparisons were performed using Dunn's (1964) procedure with a Bonferroni correction for multiple comparisons; for *t* tests the adjusted *P* value was therefore 0.013, and for the partial correlations it was 0.003.

All analyses were carried out using SPSS® version 20.0 (IBM, Armonk, New York, USA) on a data set available on Zenodo^28^.

## Results

The participants were 44 core MDT members (breast, 15; colorectal, 15; and gynaecological, 14). The MDTs had the same composition: surgeons (12), oncologists (6), CNSs (12), radiologists (6), histopathologists (5), and coordinators (3). Allied health professionals were not included. In total, the MDTs discussed 822 patients across 30 MDT meetings during the study.

### Descriptive statistics

*Table 1* provides an overview of the MDT meeting characteristics. The gynaecological MDT had the highest workload and longest meetings, whereas the colorectal team had the least number of cases and shortest meeting duration. The colorectal team also spent most time discussing each patient, followed closely by the gynaecological and breast teams. In terms of team composition, breast and colorectal teams had similar number of members attending the meetings; the gynaecological team was the smallest. There were more women in attendance in breast and colorectal teams, whereas in the gynaecological team there were more men.

**Table 1:** Meeting characteristics of breast, colorectal, and gynaecological cancer team meetings

	*M*	s.d.	Min	Max
**Overall (*n* = 818)**				
Meetings observed	30	–	–	–
Case discussions observed	822	–	–	–
Case discussions per meeting	33	11	15	51
Meeting duration (hours:minutes)	01:55	01:00	00:40	04:00
Time per patient (minutes:seconds)	01:34	02:04	00:06	15:23
Core MDT members present	9	3	4	15
Females* (%)	52	–	–	–
Males† (%)	48	–	–	–
**Breast team (*n* = 241)**				
Meetings observed	10	–	–	–
Case discussions observed	241	–	–	–
Case discussions per meeting	26	3	20	30
Meeting duration (hours:minutes)	01:06	00:12	00:52	01:31
Time per patient (minutes:seconds)	02:25	01:56	00:06	10:19
Core MDT members present	11	2	5	15
Females* (%)	64	–	–	–
Males† (%)	36	–	–	–
**Colorectal team (*n* = 185)**				
Meetings observed	10	–	–	–
Case discussions observed	185	–	–	–
Case discussions per meeting	20	4	15	27
Meeting duration (hours:minutes)	01:00	00:15	00:40	01:30
Time per patient (minutes:seconds)	03:02	02:20	00:12	14:02
Core MDT members present	11	2	5	15
Females* (%)	57	–	–	–
Males† (%)	43	–	–	–
**Gynaecological team (*n* = 392)**				
Meetings observed	10	–	–	–
Case discussions observed	396	–	–	–
Case discussions per meeting	43	5	35	51
Meeting duration (hours:minutes)	02:52	00:35	01:57	04:00
Time per patient (minutes:seconds)	02:30	01:57	00:06	15:25
Core MDT members present	7	1	4	10
Females* (%)	33	–	–	–
Males† (%)	67	–	–	–

Reprinted with permission from Soukup, 2017^29^. *M*, mean; MDT, multidisciplinary team.

* Females (*n* = 27): 3 surgeons, 4 oncologists, 2 pathologists, 11 cancer nurse specialists, 4 radiologists, 3 MDT coordinators.

†Males (*n* = 17): 9 surgeons, 3 radiologists, 2 oncologists, 2 pathologists, 1 cancer nurse specialist.

*Table 2* shows descriptive statistics for the composite score of each measure used in the study. The colorectal team had the highest mean scores on all three measures, with the most intensified interaction process and most complex case discussions. The breast team closely followed with the scores on the interaction process; however, both breast and gynaecological teams had similar mean scores for decision-making quality and case complexity.

**Table 2:** Descriptive statistics for the composite scores of the Measure of Discussion Complexity (MeDiC), Metric for Observation of Decision-making (MDT-MODe), and Bales' Interaction Process Analysis (Bales' IPA)

Instrument (score range)	MeDiC (0 to infinity‡)	MeDiC (0 to infinity‡)	MODe (11 to 55*)	Bales' IPA (0 to infinity†)
Measuring	Logistical issues	Clinical complexity	Decision-making	Communication
**Overall (*n* = 818)**				
Mean(s.d.)	0.5(0.7)	4.1(3.8)	23.8(6.0)	26.1(17.9)
Median (i.q.r.)	0 (1)	3 (5)	23 (9)	22 (18)
Min–max	0–3	0–26	11–44	4–99
**Breast team (*n* = 241)**				
Mean(s.d.)	0.4(0.7)	3.7(3.6)	23.3(6.6)	28.6(20.8)
Median (i.q.r.)	0 (1)	3 (4)	23 (10)	23 (28)
Min–max	0–2	0–18	11–44	4–99
**Colorectal team (*n* = 185)**				
Mean(s.d.)	0.9(0.8)	6.2(3.8)	25.6(5.9)	29.1(18.3)
Median (i.q.r.)	1 (1)	6 (5)	26 (7)	25 (22)
Min–max	0–3	0–19	11–42	4–96
**Gynaecological team (*n* = 392)**				
Mean(s.d.)	0.4(0.6)	3.4(3.6)	23.2(5.6)	23.1(15.1)
Median (i.q.r)	0 (1)	2 (3)	23 (8)	19 (18)
Min–max	0–3	0–16	11–42	4–99

Reprinted with permission from Soukup, 2017^29^. MeDiC, Measure of Discussion Complexity; MODe, Metric for Observation of Decision-making; IPA, Bales Interaction Process Analysis; i.q.r., interquartile range.

* Composite MODe score is a sum of 11 individual variables each scored on a range of 1 to 5 with higher scores indicating better quality.

†Composite Bales' IPA score is a sum of 12 variables each scored as a frequency count with higher scores indicating more interactions.

‡Composite MeDiC score is a sum of 26 (binary) clinical variables and the frequency counts of logistical issues with higher scores indicating more complex case discussions.

### Reliability of evaluations

Inter-assessor agreement was examined in a subset of the observed cases: 136 (17 per cent) for MeDiC; 158 (20 per cent) for MDT-MODe; and 117 (15 per cent) for Bales' IPA. For the composite values across the tools, reliability was as follows: ICC = 0.995 (95 per cent c.i 0.994 to 0.997) for MeDiC; ICC = 0.934 (95 per cent c.i. 0.909 to 0.952) for MODe; and ICC = 0.993 (95 per cent c.i. 0.989 to 0.996) for Bales' IPA tool.

### Frequency of logistical challenges across the discussed cases

The thematic analysis carried out including the description and frequency of each type and instance of logistical challenges are in *Table 3*. The most frequent logistical problems were administrative and process issues that related to the pathology and radiology results not being ready; insufficient detail on patient referral/request forms; unavailability of patient clinical records; clarity as to why the patient was included in the MDT list; and issues around outsourcing tests and non-standardized forms.

**Table 3:** Results from thematic analysis with definitions and frequencies of logistical challenges across the cancer cases (presented in order of item frequency)

Discourse and dimension	Frequency across cases
**Administrative errors and process issues**	238/397 (30)
Radiology (42) or pathology (81) results not ready or not yet done	123/238 (52)
Insufficient details on request/referral forms or reports from other hospital, MDT, or GP	55/238 (23)
Patient notes are missing/not available at the point of the meeting	36/238 (15)
Team is not sure why is the patient on MDT list or why certain tests were performed	23/238 (10)
Issues with outsourcing tests and non-standardized forms so some information or results are missing or delayed, and need to be chased up	20/238 (8)
There are issues with appointments and who is going to follow-up with the patient due to overbooking	14/238 (6)
Side of lesion is mixed up	7/238 (3)
There were problems with diagnostic equipment, so tests were not done in time for the MDT	6/238 (2.5)
Patient's DOB or name spelling is incorrect and so their radiology images or pathology results cannot be found	3/238 (1)
One of the core members needs to leave the meeting to obtain missing information/report	1/238 (0.5)
**Attendance issues**	121/397 (16)
One of the core members that is needed to make a decision is not present so decision cannot be reached at this point and case needs to be re-discussed when the member arrives. There is no radiologist (or they are running late) and so patients that need radiology input cannot be discussed, which leads to them being discussed again later in the meeting (twice), or those that need oncologist input may need to be re-discussed again later if the oncologist is not there, or the responsible clinician is not around and the team feels that they are not able to make a treatment plan until they arrive	107/121 (88)
No one present has seen the patient, and so there is insufficient information to make treatment plan and the patient needs to be re-discussed the following week	40/121 (33)
**Issues with meeting equipment**	38/397 (5)
Team is not able to connect with another site (such as using videoconferencing), which provides input from disciplines and specialties that are not able to be physically present in the meetings; this means that the discussion for patients needing the input from them is delayed and will need to be repeated later in the meeting or next week	38/38 (100)
Slides are not working and so pathology and imaging cannot be shown to the team	2/38 (5)
Computer system is slow or not working and so patient information (such as written pathology report) cannot be accessed or retrieved, and so the patient needs to be postponed for the following week	1/38 (3)

Values are *n* (%) unless otherwise indicated. Some cases have more than one logistical issue (one logistical issue per discussion occurred in 32 per cent of cases, two logistical issues occurred in 7 per cent of cases, three logistical issues occurred in 2 per cent of cases, and four logistical issues occurred in 0.1 per cent of cases). Reprinted with permission from Soukup, 2017^29^. MDT, multidisciplinary team; GP, general practitioner; DOB, date of birth.

Across the reviewed cancer cases, the frequency of administrative errors and process issues was higher (238 of 818 cases) than the frequency of equipment issues (38 cases), *t*(818) = 16.84, *P* < 0.001, and the frequency of attendance issues (121 cases), *t*(818) = 11.32, *P* < 0.001. The frequency of attendance issues was higher than the frequency of equipment issues, *t*(818) = 6.31, *P* < 0.001. The same pattern was also evident for each of the participating MDTs individually, although for breast and gynaecological MDTs, the statistical significance was not reached (all *P* > 0.013) for the comparison between the frequency of equipment issues (2 of 241 cases and 0 of 392 cases respectively) against the frequency of administrative and process issues (80 and 62 cases respectively), and the frequency of attendance issues (14 and 62 cases respectively; *Table 4*).

**Table 4:** Descriptive statistics for the logistical challenges across teams and overall data set

Logistical challenges	Admin and process issues	Attendance issues	Equipment issues	Overall issues
**Overall (*n* = 818)**				
Mean(s.d.)	0.34(0.58)	0.16(0.41)	0.05(0.21)	0.53(0.73)
Number of cases with an issue	238 (30)	121 (16)	38 (5)	397 (51)
Average number of issues per case	1 (24)	1 (13)	1 (5)	1 (42)
Min–max number of issues per case	0–3	0–3	0–1	0–3
**Breast team (*n* = 241)**				
Mean(s.d.)	0.38(0.57)	0.07(0.28)	0.01(0.09)	0.44(0.66)
Number of cases with an issue	80 (33)	14 (6)	2 (1)	96 (40)
Average number of issues per case	1 (29)	1 (5)	1 (1)	3 (35)
Min–max number of issues per case	0–2	0–2	0–1	0–2
**Colorectal team (*n* = 185)**				
Mean(s.d.)	0.41(0.64)	0.35(0.50)	0.19(0.40)	0.90(0.84)
Number of cases with an issue	62 (34)	62 (34)	36 (20)	160 (88)
Average number of issues per case	1 (27)	1 (32)	1 (20)	3 (79)
Min–max number of issues per case	0–3	0–2	0–1	0–3
**Gynaecological team (*n* = 392)**				
Mean(s.d.)	0.29(0.56)	0.14(0.41)	0(0)	0.41(0.65)
Number of cases with an issue	96 (25)	45 (12)	0	141 (37)
Average number of issues per case	1 (21)	1 (9)	0	2 (30)
Min–max number of issues per case	0–3	0–3	0	0–3

Values are *n* (%) unless otherwise indicated. *n* = 818 cases (19 missing cases).

### Relationships between the types of logistical challenges and the quality of decision-making and communication

*Table 5* shows the results of the partial correlation analysis controlling for the clinical complexity of cases. The relationship between the administrative errors and process issues and the quality of information was significantly negative, whereas a significant positive relationship was evident with the frequency of asking questions and providing answers. Some variation in its relationship with negative reactions was evident across the teams; however, with the breast cancer MDT showing negative correlations and the gynaecological cancer team showing positive correlations. The relationship between equipment issues and quality of discussions, and positive reactions was negative. There was a correlation between attendance issues and the quality of information and positive reactions.

**Table 5:** Results from partial correlation analysis between logistical challenges and the quality of multidisciplinary team decision-making and communication while controlling for clinical complexity of cases across the three cancer teams

Logistical challenges	Admin and process issues		Equipment issues		Attendance issues	
**Overall (*n* = 818)**						
Decision-making	*r*	*P*	*r*	*P*	*r*	*P*
Quality of information	**−0**.**15**	**0**.**001**	0.04	0.208	**−0**.**11**	**0**.**001**
Quality of discussion	−0.00	0.926	**−0**.**14**	**0**.**003**	−0.03	0.405
Communication						
Asking questions (task-oriented)	**0**.**21**	**0**.**001**	−0.05	0.219	−0.05	0.405
Providing answers (task-oriented)	**0**.**19**	**0**.**001**	0.04	0.138	0.02	0.606
Positive socioemotional reactions	**0**.**14**	**0**.**001**	−0.08	0.026	−0.11	0.026
Negative socioemotional reactions	**0**.**14**	**0**.**001**	0.02	0.485	−0.06	0.485
**Breast team (*n* = 241)**						
Decision-making						
Quality of information	−0.15	0.021	0.03	0.695	−0.07	0.312
Quality of discussion	−0.03	0.631	0.04	0.522	−0.08	0.239
Communication						
Asking questions (task-oriented)	0.13	0.049	−0.04	0.516	−0.02	0.808
Providing answers (task-oriented)	**0**.**24**	**0**.**001**	0.04	0.594	0.05	0.440
Positive socioemotional reactions	0.3	0.635	−0.07	0.318	−0.09	0.148
Negative socioemotional reactions	**0**.**17**	**0**.**001**	0.05	0.450	−0.08	0.221
**Colorectal team (*n* = 185)**						
Decision-making						
Quality of information	**−0**.**20**	**0**.**003**	−0.15	0.116	0.08	0.310
Quality of discussion	−0.09	0.242	−0.21	0.005	0.09	0.208
Communication						
Asking questions (task-oriented)	0.19	**0**.009	−0.12	0.116	0.12	0.094
Providing answers (task-oriented)	0.16	0.033	0.14	0.061	−0.21	0.005
Positive socioemotional reactions	0.17	0.020	−0.03	0.680	0.09	0.091
Negative socioemotional reactions	0.12	0.095	−0.07	0.319	0.01	0.876
**Gynaecological team (*n* = 392)**						
Decision-making						
Quality of information	**−0**.**15**	**0**.**003**	N/A	N/A	**−0**.**18**	**0**.**001**
Quality of discussion	0.02	0.749	N/A	N/A	−0.10	0.050
Communication						
Asking questions (task-oriented)	**0**.**26**	**0**.**001**	N/A	N/A	−0.14	0.040
Providing answers (task-oriented)	**0**.**19**	**0**.**001**	N/A	N/A	−0.10	0.040
Positive socioemotional reactions	**0**.**20**	**0**.**001**	N/A	N/A	−0.11	0.039
Negative socioemotional reactions	0.03	0.532	N/A	N/A	−0.02	0.645

*n* = 818 (19 missing cases). Bonferroni-adjusted significance level is 0.003. *r* = partial correlation coefficient (controlling for case complexity). **Bold** indicates significant coefficients. N/A, not available.

## Discussion

The aim of the study was to understand the frequency with which logistical challenges occurred in MDT meetings, and how these issues affected the MDTs' decision-making and communication. The study found that the most frequent were administrative and process issues relating to pathology and radiology results not being ready, preventing the MDT from formulating treatment plans and leading to case discussions being postponed. This was closely followed by a lack of sufficient information on request and referral forms from other hospitals, general practice, or other MDTs; deficient or missing medical records at the time of the meeting; uncertainties regarding the reason for listing the case for MDT discussion; and issues around outsourcing tests and non-standardized forms leading to information or results not being available. Other administrative and process issues around appointments, availability of diagnostic equipment, incorrect site or side of a lesion, and errors in patients' personal details were also identified, but these occurred at a markedly lower rate. The other major logistical issue was related to meeting attendance, notably when the core member needed to make a specific decision was absent, or when no one present has first-hand knowledge of the patient. The least frequent of the three major logistical challenges were those around meeting equipment, in particular the inability to connect with another site for input, difficulty displaying pathology/radiology information to the team, and difficulty retrieving patient information from electronic systems.

The hypothesis that all types of logistical issues would negatively relate to the quality of decision-making, and positively to communication, intensifying the interaction process, was therefore largely supported, with a few exceptions. The relationship between types of logistical issues and communication and decision-making in the meetings was more complex than anticipated. As the frequency of administrative errors and process issues, as well as attendance issues increased, the quality of patient information decreased, as expected. On the other hand, there was no relationship between the frequency of the above issues and the quality of contribution, except for equipment issues, which showed the same negative trend.

As seen in both the present study and previous research^24,25^, logistical issues intensified task-oriented communication and socioemotional interactions between team members—arguably to rectify errors and compensate for issues such as technical failures or lack of attendance of key members^24,25,35^. In particular, more questioning and answering was evident as a result of administrative and process issues. Increased socioemotional reactions were also evident with some teams displaying an increase in positive (gynaecological cancer MDTs) and others in negative reactions (breast cancer MDT). Similarly, equipment and attendance issues were associated with a decrease in positive reactions, with some variation evident across teams. Most notably, in the gynaecological cancer MDT, attendance issues were associated with poorer quality of decision-making, whereas in the colorectal team they were associated with reduced task-oriented communication in terms of providing answers to queries.

The present findings are important as healthcare teams are frequently constrained by financial pressures^16,17^, staff shortages^19^, increasing cancer incidence^16,18^, growing workload^20^, and various human factors^21–25^. Understanding what impacts the performance of an MDT and how it can be made more efficient is critical to quality improvement^36,37^. Some issues could be addressed ahead of the meeting, using the MeDiC tool for example^24,25,32^ so that the impacts of these logistical challenges cause less upset to the team dynamics and delays in care.

Several strategies were used to strengthen the validity and generalizability of the study along with efforts to reduce the risks of bias. Attempts to minimize the Hawthorne effect involved a long-term approach by filming each team for a prolonged interval, excluding the first two meetings in each team from the analysis and ensuring that filming was carried out discretely. Validated observational tools scored by trained evaluators in pairs blind to one another's observations were also used to reduce bias. The sample size was adequate for an observational study, and the chosen cancers represented the most common cancers within the English NHS. Despite these measures, the present study has limitations. While an observational approach allowed the capture of complex organizational behaviour in cancer MDTs in real time (providing good external validity and identifying new avenues of research), the replication of the study for other cancers, teams, and healthcare systems is still needed to determine generalizability of the findings. This seems particularly important in relation to attendance issues, which seemed to be the driver of logistical challenges in the colorectal MDT. The present study was focused on decision-making processes at the point of the MDT meeting and no attempt has been made to link these to clinical, patient-related outcomes. As a result, clinical implications of this analysis require further study. This work might well disclose additional issues not picked up by the present study.

Despite these shortcomings, the present study has identified that logistical challenges are common problems within cancer MDTs, reducing the quality of decision-making and intensifying the communication process.

## Acknowledgements

T.S. made a substantial contribution to the conception and design of the study. T.S., B.W.L., N.S. and J.S.A. made substantial contributions to the analysis and interpretation of data. A.D., N.S., and J.S.A.G. contributed equally and share senior authorship. All authors made substantial contributions to the acquisition of data, drafting the manuscript, and revising it critically for important intellectual content, and have given final approval of the version to be published, and have agreed to be accountable for all aspects of the work in ensuring that questions related to the accuracy or integrity of any part of the work are appropriately investigated and resolved. The authors thank the cancer MDTs and their members for their time and commitment to the project. The study was granted ethical and regulatory approvals by the North West London Research Ethics Committee (JRCO ref. 157441), and also locally by the R&D departments of the participating NHS Trusts. Informed consent was sought from all participants. The study was adopted by the National Institute for Health Research (NIHR) Clinical Research Network Portfolio.

## Supplementary Material

zrac093_Supplementary_Data

## Data Availability

The anonymized data set supporting this study is available in Zenodo, a research data repository (https://zenodo.org/record/582272#.XntHvoj7Q2w) under the Creative Commons Attribution Non-Commercial Non-Derivative 4.0 license. Researchers are free to reuse and redistribute the data set on the condition that they attribute it, that they do not use it for commercial purposes, and that they do not alter it. For any reuse or redistribution, researchers must make clear to others the license terms of this work and reference the data set accordingly.
