# Supplementary material for: Cancer multidisciplinary team meetings: impact of logistical challenges on communication and decision-making
Source: BJS Open. 2022 Aug 27;6(4):zrac093. doi: 10.1093/bjsopen/zrac093 (PMC9418925; doi:10.1093/bjsopen/zrac093)
Supplement: zrac093_Supplementary_Data [file zrac093_supplementary_data.zip › Supplementary_Table_2.docx]

## Table S2. Description of the tools used in the study

| **METRIC FOR OBSERVATION OF DECISION-MAKING (MODE)** |
| --- |
| **Decision-making process was assessed using the Metric for Observation of Decision-making, (MODe)**. MODe has been used previously to assess various cancer MDT meetings and has shown good validity and reliability. It captures the following aspects:   1. **Quality of presented patient information**, which includes 6 variables scored on a behaviourally anchored 5-point scale, namely, patients’ case history, radiological images, histopathology, psychosocial issues, co-morbidities and their views on treatment options. The sum of the scores for all 6 variables represents overall quality of presented information for a patient with the higher scores indicating better quality. 2. **Quality of contribution to case-reviews**, which includes 6 variables scored on a behaviourally anchored 5-point scale, representing contributions made by the surgeons, oncologists, radiologists, histopathologists, cancer nurse specialist and the chair-person of the meeting. The sum of the scores for all 6 variables represents overall quality of contribution for a patient with the higher scores indicating better quality. |
| **MEASURE OF CASE-DISCUSSION COMPLEXITY (MEDIC)** |
| **Complexity of each patient discussed in the meeting was assessed using a psychometrically valid and reliable tool, namely, Measure of case-Discussion Complexity (MeDiC).** MeDiC has been developed following a multiphase research process over 18 months with input from cancer specialists throughout at national level in the UK. It demonstrated evidence of reliability and validity in its scores, as well as feasibility in utilisation by both medically and non-medically trained assessors. MeDiC captures clinical complexity (incl. pathology, patient factors and treatment factors), and logistical complexity (administrative and process of care issues) for each patient discussed in the MDT meeting – the former is scored using a checklist principle (with added weight for certain items), while the latter is scored as frequency (tally for every occurrence). |
| **BALES INTERACTION PROCESS ANALYSIS (BALES IPA)** |
| **Interaction process between team members was assessed using Bales Interaction Process Analysis (Bales IPA)**. This is an observational coding system developed initially with small health care teams engaged in weekly diagnostic meetings at Harvard Psychological clinic, and further refined in simulated team meetings. It is based on a principle that a small group represents individuals (2 to 20 people) engaged in a face to face interaction (in a meeting or series of such meetings) where basic formal similarities irrespective of the context and inherent values exist, i.e. “certain types of action tend to have certain types of effects on subsequent action”. As such, it is particularly suitable for cancer MDT meetings: while it was developed and validated within a very similar setting i.e. within weekly health care team meetings, it can be used in groups that are diverse in composition, character and purpose (e.g., diagnostic and policy forming committees, boards and panels, group therapy and training, work groups, doctor-patient dyads). For every patient discussed in the meeting, four aspects of MDT interaction were captured using frequency counts by marking the originator and target of each interaction while following the specific rules and framework; this is as follows:  **Positive reactions (socio-emotional area)**   1. Shows solidarity, cooperation, gives help, raises others status, friendly; 2. Tension release, jokes, laughs, shows satisfaction; 3. Agrees, shows passive acceptance, understands, complies, concurs;   **Giving answers (task-directed area)**   1. Gives suggestion, direction, instruction, solution, way to achieve goal; 2. Gives opinion, evaluation, interpretation, decision-making, reasoning; 3. Gives orientation, information, repeats, confirms, clarifies;   **Asking questions (task-directed area)**   1. Asks for orientation, information, repetition, confirmation, clarification; 2. Asks for opinion, evaluation, interpretation, decision-making, reasoning; 3. Asks for suggestion, direction, instruction, solution, way to achieve goal;   **Negative reactions (socio-emotional area)**   1. Disagrees, shows passive rejection, un-acknowledging (e.g. doing something other than the task such as whispering), hesitant, critical, withholds help; 2. Shows tension, fear of provoking opposition, frustrated, concerned, asks for help; 3. Shows antagonism, deflates other’s status, asserts self, autocratic. |
