# Supplementary material for: Cancer multidisciplinary team meetings: impact of logistical challenges on communication and decision-making
Source: BJS Open. 2022 Aug 27;6(4):zrac093. doi: 10.1093/bjsopen/zrac093 (PMC9418925; doi:10.1093/bjsopen/zrac093)
Supplement: zrac093_Supplementary_Data [file zrac093_supplementary_data.zip › Supplementary_Table_3.docx]

## Table S3a. Descriptive Statistics for Individual Items of the Measure of case-Discussion Complexity, MeDiC

| **MeDiC** Items | **Breast team** (*n* = 241) | **Colorectal team** (*n* = 185) | **Gynaecological team** (*n* = 396) | **Overall** (*n*= 822) |
| --- | --- | --- | --- | --- |
|  | Sum | Sum | Sum | Sum |
| 1. Malignancy | 113 | 124 | 204 | 441 |
| 2. Invasive component | 80 | 81 | 90 | 251 |
| 3. Residual tumour | 13 | 9 | 25 | 47 |
| 4. Recurrence | 5 | 10 | 28 | 43 |
| 5. Multiple cancers | 21 | 20 | 27 | 68 |
| 6. Increased size (T3, T4) | 18 | 51 | 11 | 80 |
| 7. Nodes affected | 32 | 43 | 28 | 103 |
| 8. Mets (local or distant) | 32 | 39 | 39 | 110 |
| 9. Advanced stage, progressive | 28 | 25 | 49 | 102 |
| 10. Unusual or rare tumour type | 9 | 11 | 14 | 34 |
| 11. Previous history of cancer | 21 | 31 | 37 | 89 |
| 12. Previous oncological treatments | 16 | 21 | 10 | 47 |
| 13. Significant surgical history | 21 | 34 | 27 | 82 |
| 14. Significant physical comorbidity | 20 | 42 | 52 | 114 |
| 15. Mental health/ cognitive comorbidity | 3 | 8 | 2 | 13 |
| 16. Socio-economic issues | 2 | 1 | 0 | 3 |
| 17. Lifestyle risks | 0 | 3 | 4 | 7 |
| 18. Patient choice and family opinion | 19 | 15 | 28 | 62 |
| 19. Diagnostic uncertainty | 33 | 40 | 32 | 105 |
| 20. Further tests and patient assessment needed | 63 | 75 | 97 | 235 |
| 21. Further input needed from other specialties | 22 | 39 | 54 | 115 |
| 22. Unusual anatomy/ distribution of tumour | 2 | 22 | 12 | 36 |
| 23. Guidelines do not account for patients situation | 1 | 0 | 0 | 1 |
| 24. Conflict of opinions about treatment options | 21 | 3 | 23 | 47 |
| 25. Treatment toxicity and contraindications | 2 | 1 | 2 | 5 |
| 26. Trial eligibility | 1 | 1 | 2 | 4 |
|  | ***M* (*SD*)** | ***M* (*SD*)** | ***M* (*SD*)** | ***M* (*SD*)** |
| 27. Logistical complexity (frequency count) | 2.52 (2.5) | 4.05 (2.5) | 2.26 (2.4) | 2.74 (2.6) |
| Total clinical complexity (sum of items 1 to 26) | 4 (4) | 6 (4) | 3 (4) | 4 (4) |
| Total complexity (sum of clinical and logistical) | 2.96 (2.7) | 4.95 (2.7) | 2.67 (2.5) | 3.27 (2.7) |

*Note.* All scores are frequency counts conducted per patient-discussion within each meeting. MeDiC = Measure of Discussion Complexity. Reprinted with permission from Soukup T. Socio-cognitive factors that affect decision-making in cancer multidisciplinary team meetings [PhD Thesis; Clinical Medicine Research]. Imperial College London. London, UK; 2017.

## Table S3b. Descriptive Statistics for Individual Items of the Metric for the Observation of Decision-Making in Cancer Multidisciplinary Teams, MDT-MODe

| **MDT-MODe Items** | **Breast team**  (*n* = 241) | | | **Colorectal team**  (*n* = 185) | | | **Gynaecological team**  (*n* = 396) | | | **Overall**  (n = 822) | | | |
| --- | --- | --- | --- | --- | --- | --- | --- | --- | --- | --- | --- | --- | --- |
|  | *M* (*SD*) | *Mdn* (*IQR*) | Min, Max | *M* (*SD*) | *Mdn* (*IQR*) | Min, Max | *M* (*SD*) | *Mdn* (*IQR*) | Min, Max | *M* (*SD*) | *Mdn* (*IQR*) | Min, Max |  |
| 1. Patient history | 2.56 (3) | 3 (1) | 1, 4 | 2.64 (0.78) | 3 (1) | 1, 5 | 2.51 (0.82) | 2 (1) | 1, 5 | 2.55 (0.79) | 3 (1) | 1, 5 |  |
| 2. Radiology | 2.50 (1.73) | 1 (3) | 1, 5 | 3.83 (1.78) | 5 (4) | 1, 5 | 3.67 (1.79) | 5 (4) | 1, 5 | 3.36 (1.86) | 4 (4) | 1, 5 |  |
| 3. Histopathology | 3.42 (1.24) | 4 (0) | 1, 5 | 2.04 (1.48) | 1 (3) | 1, 5 | 2.44 (1.74) | 1 (3) | 1, 5 | 2.64 (1.64) | 3 (3) | 1, 5 |  |
| 4. Psychosocial | 1.12 (0.48) | 1 (0) | 1, 4 | 1.16 (0.57) | 1 (0) | 1, 5 | 1.05 (0.29) | 1 (0) | 1, 3 | 1.09 (0.43) | 1 (0) | 1, 5 |  |
| 5. Comorbidities | 1.15 (0.55) | 1 (0) | 1, 4 | 1.23 (0.69) | 1 (0) | 1, 5 | 1.17 (0.70) | 1 (0) | 1, 5 | 1.18 (0.66) | 1 (0) | 1, 5 |  |
| 6. Patient views | 1.18 (0.75) | 1 (0) | 1, 5 | 1.11 (0.53) | 1 (0) | 1, 5 | 1.07 (0.44) | 1 (0) | 1, 5 | 1.11 (0.57) | 1 (0) | 1, 5 |  |
| Information score  (sum of 1 to 6)* | 11.92 (2.65) | 11 (4) | 6, 23 | 12.01 (2.73) | 12 (3) | 6, 25 | 11.91 (2.50) | 11 (2) | 6, 22 | 11.93(2.6) | 12 (2) | 6, 25 |  |
| 7. Surgeons’ input | 3.57 (1.54) | 4 (2) | 1, 5 | 4.21 (1.29) | 5 (4) | 1, 5 | 3.69 (1.36) | 4 (2) | 1, 5 | 3.77 (1.42) | 4 (2) | 1, 5 |  |
| 8, Oncologists’ input | 2.27 (1.79) | 1 (4) | 1, 5 | 2.25 (1.77) | 1 (4) | 1, 5 | 1.51 (1.23) | 1 (0) | 1, 5 | 1.90 (1.59) | 1 (2) | 1, 5 |  |
| 9. Nurses’ input | 1.45 (1.18) | 1 (0) | 1, 5 | 2.46 (1.72) | 1 (4) | 1, 5 | 1.36 (1.02) | 1 (0) | 1, 5 | 1.63 (1.33) | 1 (0) | 1, 5 |  |
| 10. Radiologists’ input | 1.80 (1.48) | 1 (0) | 1, 5 | 2.92 (1.88) | 3 (4) | 1, 5 | 2.48 (1.74) | 1 (3) | 1, 5 | 2.38 (1.75) | 1 (3) | 1, 5 |  |
| 11. Pathologists’ input | 2.32 (1.73) | 1 (3) | 1, 5 | 1.74 (1.45) | 1 (0) | 1, 5 | 2.24 (1.74) | 1 (3) | 1, 5 | 2.15 (1.69) | 1 (3) | 1, 5 |  |
| Contribution score  (sum of 7 to 11)* | 11.41 (4.77) | 11 (6) | 5, 25 | 13.59 (4.23) | 13 (6) | 5, 23 | 11.29 (3.79) | 11 (5) | 5, 25 | 11.84 (4.3) | 12 (6) | 5, 25 |  |

*Note.* *Information score ranges from 5 to 30. †Contribution score from 5 to 25. Individual items are scored on a range from 1 to 5 with higher scores indicating better quality. Reprinted with permission from Soukup T. Socio-cognitive factors that affect decision-making in cancer multidisciplinary team meetings [PhD Thesis; Clinical Medicine Research]. Imperial College London. London, UK; 2017.

## Table S3c. Descriptive Statistics for Individual Items of the Bales’ Interaction Process Analysis, Bales’ IPA

| **Bales’ IPA Items** | **Breast team**  (*n* = 241) | | | **Colorectal team**  (*n* = 185) | | | **Gynaecological team**  (*n* = 396) | | | **Overall**  (*N* = 822) | | |
| --- | --- | --- | --- | --- | --- | --- | --- | --- | --- | --- | --- | --- |
|  | *M* (*SD*) | *Mdn* (*IQR*) | Min,  Max | *M* (*SD*) | *Mdn* (*IQR*) | Min, Max | *M* (*SD*) | *Mdn* (*IQR*) | Min, Max | *M* (*SD*) | *Mdn* (*IQR*) | Min, Max |
| 1. Shows solidarity | 0.56 (0.99) | 0.00 (1) | 0, 5 | 0.31 (0.77) | 0.00 (0) | 0, 5 | 0.27 (0.67) | 0.00 (0) | 0, 6 | 0.37 (0.81) | 0.00 (0) | 0, 6 |
| 2. Tension release | 0.91 (1.45) | 0.00 (1) | 0, 7 | 0.30 (0.70) | 0.00 (0) | 0, 3 | 0.86 (1.52) | 0.00 (1) | 0, 9 | 0.75 (1.37) | 0.00 (1) | 0, 9 |
| 3. Agrees | 3.30 (3.56) | 2 (4) | 0, 26 | 2.18 (2.31) | 2 (3) | 0, 12 | 1.37 (1.77) | 1 (2) | 0, 12 | 2.12 (2.66) | 1 (3) | 0, 26 |
| Positive reactions (sum of 1 to 3) | 4.77 (4.79) | 4 (6) | 0, 32 | 2.8 (2.96) | 2 (3) | 0, 14 | 2.51 (2.58) | 2 (3) | 0, 14 | 3.24 (3.58) | 2 (3) | 0, 32 |
| 4. Gives suggestion | 3.40 (1.95) | 3 (2) | 1, 12 | 2.81 (1.59) | 2 (2) | 1, 11 | 2.72 (1.75) | 2 (2) | 0, 11 | 2.94 (1.8) | 2 (2) | 0, 12 |
| 5. Gives opinion | 5.73 (6.79) | 3 (8) | 0, 38 | 4.40 (5.44) | 2 (5) | 0, 30 | 3.24 (4.72) | 1 (5) | 0, 26 | 4.23 (5.65) | 2 (6) | 0, 38 |
| 6. Gives suggestion | 5.10 (4.27) | 4 (5) | 0, 23 | 7.70 (6.08) | 7 (8) | 0, 30 | 7.44 (5.31) | 7 (7) | 0, 28 | 6.82 (5.33) | 6 (7) | 0, 30 |
| Gives answers  (sum of 3 to 6) | 14.23 (10.7) | 11 (12) | 2, 60 | 14.91 (10.7) | 12 (10.5) | 2, 60 | 13.40 (9.21) | 11 (9.75) | 1, 56 | 13.98 (10.0) | 11 (11) | 1, 60 |
| 7. Asks for orientation | 3.84 (4.31) | 2 (5) | 0, 21 | 5.55 (4.86) | 5 (5) | 0, 31 | 4.77 (4.44) | 4 (4) | 0, 30 | 4.67 (4.54) | 4 (5) | 0, 31 |
| 8. Asks for opinion | 1.46 (2.18) | 0.00 (2) | 0, 10 | 1.79 (2.87) | 1 (3) | 0, 19 | 0.96 (2.43) | 0.00 (1) | 0, 19 | 1.29 (2.49) | 0.00 (2) | 0, 19 |
| 9. Asks for suggestion | 0.36 (0.71) | 0.00 (1) | 0, 3 | 0.28 (0.63) | 0.00 (0) | 0, 4 | 0.06 (0.43) | 0.00 (0) | 0, 7 | 0.20 (0.58) | 0.00 (0) | 0, 7 |
| Asks questions  (sum of 6 to 9) | 5.66 (5.99) | 4 (8) | 0, 30 | 7.61 (7.47) | 6 (7) | 0, 50 | 5.79 (6.59) | 4 (5) | 0, 51 | 6.16 (6.67) | 4 (6) | 0, 51 |
| 10. Disagrees | 2.29 (2.72) | 1 (4) | 0, 13 | 1.68 (2.05) | 1 (3) | 0, 10 | 0.87 (1.58) | 0.00 (1) | 0, 10 | 1.47 (2.17) | 0.00 (2) | 0, 13 |
| 11. Shows tension | 1.02 (2.97) | 0.00 (0) | 0, 22 | 0.97 (2.66) | 0.00 (0) | 0, 14 | 0.42 (1.29) | 0.00 (0) | 0, 7 | 0.72 (2.25) | 0.00 (0) | 0, 22 |
| 12. Shows antagonism | 0.59 (1.20) | 0.00 (1) | 0, 9 | 1.12 (1.46) | 1 (2) | 0, 6 | 0.13 (0.67) | 0.00 (0) | 0, 8 | 0.49 (1.29) | 0.00 (0) | 0, 9 |
| Negative reactions (sum of 10 to 12) | 3.9 (4.15) | 3 (5) | 0, 23 | 3.77 (3.39) | 3 (5) | 0, 15 | 1.41 (2.00) | 0.00 (2) | 0, 10 | 2.67 (3.32) | 2 (4) | 0, 23 |

*Note.* All scores are frequency counts conducted per patient-discussion within each meeting. Reprinted with permission from Soukup T. Socio-cognitive factors that affect decision-making in cancer multidisciplinary team meetings [PhD Thesis; Clinical Medicine Research]. Imperial College London. London, UK; 2017.
